# Ochratoxins—Food Contaminants: Impact on Human Health

**DOI:** 10.3390/toxins2040771

**Published:** 2010-04-20

**Authors:** Lalini Reddy, Kanti Bhoola

**Affiliations:** 1 Department of Biotechnology and Food Technology, Durban University of Technology, P. O. Box 1334, Durban, 4000, South Africa; 2 University of Western Australia, The Lung Institute of Western Australia, Ground Floor E Block, Sir Charles Gairdner Hospital, Nedlands WA, 6009, Australia; Email: profbhoola@iinet.net.au

**Keywords:** ochratoxin, food, kidney disease

## Abstract

Ochratoxins are secondary metabolites of *Aspergillus* and *Penicillium*, that are hazardous to health through contamination of dietary foods. Ochratoxin A (OTA) remains the single most potent member of this group of mycotoxins. OTA has a long half-life in humans and is thus easily detected in serum. Dietary intake studies have confirmed link between endemic nephrotoxicity in humans to their daily household intake of OTA. OTA has been reported to contribute to endemic nephrotoxicity and carcinogenicity in humans and animals. OTA produces renal tumours, DNA adducts and chromosomal aberrations in kidneys. OTA may be embryotoxic, teratogenic, and immunotoxic only at doses higher than those causing nephrotoxicity. The incidence of endemic nephrotoxicity has been mostly reported in northeast Europe since the early fifties. Recent studies however have warned that OTA and other toxins, such as aristolochic acid, show very similar renal pathology. There is thus the need for thorough co-occurrence studies on toxin incidence.

## 1.Source and Chemical Structure

Ochatoxins belong to a family of mycotoxins that are secondary metabolites of *Aspergillus sp.* and *Penicillium sp*. Their growth promoting conditions are delineated in Table 1. 

**Table 1:** Growth conditions for ochratoxin production.

Growth conditions	*A. ochraceus*	*P. verrucosum*
optimum temperature for growth	24 to 37 °C	20 °C
optimum temperature for ochratoxin production	31 °C	20 °C
optimum growth pH	3 to 10	6.0 to 7.0
minimum water activity for ochratoxin production	0.8	0.86

**Figure 1:**
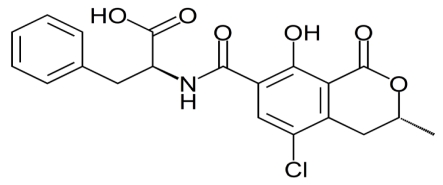
Ochratoxin A from *Aspergillus sp.*

Several types of ochratoxins occur naturally, namely, ochratoxin A, ochratoxin B (dechlorinated OTA) and ochratoxin C (ethylated OTA), and are often co-produced. Ochratoxin A (Figure 1) is the most prevalent toxin and is classified as a group 2b potential human carcinogen by the International Agency for Research on Cancer [1]. Aristolochic acids are phytotoxins belonging to the Aristolochiaceae family of plants and have been suspected to induce similar nephrotoxic effects in humans and animals [2].

## 2. Occurrence in Food and Dietary Intake

Ochratoxins occur primarily in cereals in northern Europe and Africa. A few examples of foods containing ochratoxins are listed in Table 2. 

**Table 2:** Food commodities intoxicated with ochratoxins.

Food commodity	Country
Dry beans (5–30 µg/kg)	Croatia [3]
Maize (10–50 µg/kg)	Croatia [2], Bulgaria [4]
Wheat , wheat bran	Bulgaria [4]
Barley, oats	Bulgaria [4]
Wine	South Africa [5]
Green coffee beans (18–48 μg/kg)	Congo [6]

Analysis of OTA and citrinin in food and feed samples were performed by enzyme immunoassays, which gave detection limits of 0.5 and 5 ng/g, respectively. OTA-values were confirmed by high performance liquid chromatography after immunoaffinity chromatography. Vrabcheva and co-workers [3] found the highest toxin levels in wheat, wheat bran, and oats. South African wines tested (15 white and nine red) were found to contain detectable levels (>0.01 μg/L) of OTA, with a mean of 0.16 μg/L in the white wines and a mean of 0.24 μg/L in the red wines [5]. Ochratoxins have also been found in meat, dairy and baked products, especially swine sausages and breads [7,8]. In humans a dietary intake of 1.21 µg per day has been linked to endemic nephropathy [7]. Further reports suggest that chronic dietary exposure to OTA, a mycotoxin frequently detected in various food items may be linked to the pathogenesis of endemic nephropathy, a chronic tubulointerstitial kidney disease which occurs in geographically limited areas of the Balkan region [8]. Contradictory findings have been reported by Mally and co-workers [9]. Based on food consumption data and OTA serum concentrations, they suggest that human exposure, even in areas with relatively high dietary exposure to OTA, such as endemic villages, is several orders of magnitude below doses known to cause nephrotoxicity and tumour formation in laboratory animals. 

## 3. Incidence of Disease

Ochratoxins have been related to human and animal diseases in literature especially since the early 1970s. Most reports indicate that ochratoxin plagued especially the North Eastern European countries, such as Bulgaria, Romania, Serbia, Croatia, Bosnia, Herzegovina, Slovenia, Macedonia, Monte Negro and Africa, such as Congo, South Africa, Tunisia, Morocco and Egypt [3,4,5,6,7,8,9,10,11].

## 4. Detection of Disease-Diagnosis

The concentration range of OTA in human serum is 5–50 ng/mL and OTA has a long serum half-life in various species including humans [3]. Schwerdt and others [12] measured OTA concentrations in blood of subjects suffering from various renal diseases as e.g. interstitial nephritis or mesangial proliferating glomerular nephritis and compared these values with those obtained from healthy individuals. OTA was detected in 87% of all samples. There was no significant difference between OTA concentrations of healthy individuals and patients with chronic glomerular nephritis whose sera showed OTA concentration greater than 1.5 nmol/L. In contrast, no values greater than 1.5 nmol/L were present in the sera of patients with membranous or focal-sclerotic glomerular nephritis. These preliminary results suggest that a serum concentration of OTA greater than 1.5 nmol/L may be a biomarker for glomerular nephritis. 

In May 2000, Abouzied and colleagues [7], using a competitive direct enzyme-lnked immunosorbent assay for OTA (detection limit 1 μg/kg), investigated OTA contamination in 165 samples of home-produced food (beans, potatoes, corn, wheat, flour) and feed from households in villages from the Balkan endemic nephropathy region (Vratza district) of north-western Bulgaria. Samples were collected from: (a) 20 households of Balkan endemic nephropathy villages (n = 8), and 16 households of Balkan endemic nephropathy-free households (within-village household controls) and (b) 22 Balkan endemic nephropathy-free households of Balkan endemic nephropathy-free villages and 22 households between-village controls. Balkan endemic nephropathy households consistently had a higher proportion of OTA-positive samples than within village control households, but similar (for some foods) or lower (for other foods) amounts when compared between village controls. The proportion of OTA-positive samples was also higher in household between village controls when compared to those within village controls. Furthermore, Balkan endemic nephropathy households had a similar number of OTA-positive samples when compared to the pooled values for within and between groups of households. OTA-exposure estimates, derived from our OTA-concentration findings and the reported average per capita monthly consumption of basic foods in rural Bulgaria, showed the highest OTA intake in Balkan endemic nephropathy households (1.21 μg per day), *vs*. 1.03 μg per day in between village control households, and 0.71 μg per day in within village control households. This is in agreement with other studies carried out in the same region [4,13]. These OTA intakes are higher than those reported in the European Union, and are close to the upper limits acceptable to several food-safety organizations. These data indicate that OTA may not alone cause Balkan endemic nephropathy but synergistically with other environmental toxicants and/or predisposing genotypes. Peraica *et al.* [14] recently detected OTA in food collected in the endemic areas and in blood and urine of their residents. In Serbia, a survey showed the prevalence of OTA in Balkan endemic nephropathy families associated with the presence of citrinin [15]. 

## 5. Toxicity in Animals and Humans

### 5.1. Animal toxicity

*Nephrotoxicity*: Pigs, being most sensitive to ochratoxins, suffer from porcine nephropathy. Field cases of ochratoxin-induced nephropathy in pigs have been reported from many countries. Mycotoxic porcine nephropathy is recognized as an endemic disease entity also in several northern and central European countries. Krogh and others [16] found epidemics of mycotoxic porcine nephropathy, closely related to excessive climatic conditions, to occur in pigs in the season preceding harvest. Renal damage has been induced by alimentary exposure to ochratoxin A in all single-stomach animals tested so far, including rodents, dogs, pigs and birds, and even in young ruminants still functioning as single-stomach animals. Krogh [17] states that most information on ochratoxin-induced nephropathy has been obtained in pigs during experimental studies comprising structural as well as functional changes. The renal damage is characterized morphologically by atrophy of the proximal tubules, interstitial cortical fibrosis and sclerotized glomeruli, and functionally by impairment of tubular function indicated by a reduction of maximal tubular excretion of para-aminohippurate per clearance of inulin and an increase in glucose excretion. Further, there is reduced ability to produce concentrated urine. The renal effect has been observed using exposure levels of OTA in the range 200 to 4,000 μg /kg feed [18]. 

Retrospective studies, in the absence of previous exposure data, have been performed on renal biopsy material to determine the activity of two renal tubular enzymes, phosphoenolpyruvate carboxykinase and gamma-glutamyl transpeptidase. Analysis of the activity suggested that these enzymes were sensitive indicators of OTA-induced porcine nephropathy. In pigs exposed to ochratoxin A for 1 week a 40% reduction of the enzyme activity was observed. The dose-related activity decrease of the two enzymes was accompanied by a dose-related aggravation of renal impairment, as measured by a reduction of tubular excretion of para-aminohippurate per clearance of inulin, suggesting that these enzymes are sensitive indicators of OTA-induced nephropathy [20]. Embryotoxicity, teratogenicity and immunotoxicity occur only at doses higher than those causing nephrotoxicity [20].

*Carcinogenesis*: In female pigs exposed to alimentary ochratoxin A for two years, no renal cancer was observed. OTA is metabolized and excreted relatively fast in animals, with an RL_50_ (residue elimination) in the pig of a few days for various tissues [17]. OTA is a recognized renal carcinogen in the mouse and rat [1,21,22]. The pathological lesions observed in kidneys of rats treated with OTA appear be rather different from the clinical and pathological characteristics of endemic nephropathy [19]. As soon as 1991, Pfohl-Leszkowicz *et al.* [23] demonstrated that OTA binds covalently with DNA in mice and also in rats [24]. Male rats appear to be extremely sensitive to OTA due to genetic susceptibility linked to biotransformation [25]. Recently, the chemical structure of OTA DNA adduct (C-C8dG-OTA) has been demonstrated by MS/MS [26]. 

### 5.2. Human toxicity

*Nephropathy:* In humans, ochratoxins cause endemic nephropathy, which has been described in several studies in North Eastern Europe as reported by Walker and Larsen [27,28,29,30]. Possible OTA-induced acute renal failure was recently reported in Italy after a farmer and his wife worked eight hours in a granary closed for several months. Renal biopsy in the woman who developed nonoliguric acute renal failure revealed lesions of acute tubular necrosis [31]. A strain of *Aspergillus ochraceus* producing OTA was isolated from the wheat. Chronic nephrotoxicity due to OTA is probably more usual and makes the diagnosis more difficult. Balkan endemic nephropathy, a chronic tubulointerstitial renal disease, could be due to OTA. According to Simon [32] epidemiologic studies showed that in areas where high OTA levels are reached in food and in the blood of the population, there is a high incidence of nephropathy and renal tumours. The FAO/WHO Joint Expert Committee on Food Additives indicate a provisional tolerable intake of 14.3 ng/kg bw per day, based on the LOEL for renal effects in pigs [29]. The European Scientific Committee on Food indicates a lower tolerable intake, below 5 ng/kg bw per day [29]. 

*Liver toxicity*: In man OTA exhibits unusual toxicokinetics, with a half-life in blood of 840 h (35 days) after oral ingestion. The delayed excretion of the toxin in man may be due to reabsorption during enterohepatic circulation, reabsorption from the urine after tubular secretion or extensive protein binding [33]. Since the toxin is ingested with almost every meal, humans may not be free of toxin for very long periods. Liver elimination of OTA is maintained by protein carriers that shuffle the toxin from its protein-bound form in blood into the hepatocyte and subsequently secrete the toxin into bile. The uptake carrier has been identified but less is known about the mechanism involved in the release into bile. A carrier system is also involved in the uptake of OTA by proximal tubule cells, which secrete the toxin into urine. Such systems are biological entrance gates that determine the elimination toxicokinetics of OTA and therefore have a major impact on half-life times and selective organ exposure [33].

*Carcinogenesis:* Renal tumours often occur on a dietary intake which is greater than 70 µg /kg bw per day of OTA. Individuals in the endemic region of the Balkan countries exposed to OTA developed DNA adducts in renal tissue and tumours [15,34,35]. Karyomegalic interstitial nephritis, characterized by karyomegaly in proximal and distal tubular epithelial cells, has been reported in nine patients by using a new molecular epidemiological approach [32]. Karyomegaly could be due to an environmental toxin that interferes with DNA replication. Involvement of the oxidative pathway in genotoxicity of OTA is known and oxidant stress induces DNA damage, according to Palma and others [36]. Therefore, karyomegaly could be a histological marker of interstitial nephritis due to OTA as outlined by Dietrich and Swenberg [37]. In a carcinogenic study it has been demonstrated that the mechanism by which OTA induced nephrotoxicity (notably reflected by karyomegalies) is different from cacrcinogenicity [38]. Recently Manderville and Pfohl-Leszkowicz [39] explained how oxidative pathway lead to OTA genotoxicity.

## 6. Co-Occurrence of Nephrotoxins

Studies have reported that the pathological changes produced by aristolochic acid are comparable to those produced by OTA [2]. There is therefore the need for toxin co-occurrence studies involving citrinin, fumonisin, aristolochic acid and OTA. Co-contamination of ochratoxin A and citrinin was found in varying number of households in two Balkan villages. Citrinin levels in those samples were clearly higher than those of OTA [15,35]. Data on the co-occurrence in food of OTA and other toxic mycotoxins such as citrinin and fumonisin B1 have been reported also [8,14]. Cultured cells and laboratory animals treated with combinations of OTA and other nephrotoxins, show synergistic effects [8]. The occurrence of OTA- and either citrinin or fumonisin increased genotoxicity [14,35]. 

## 7. Concluding Remarks

Despite many efforts, the aetiology of endemic nephropathy is still unknown [14]. This disease occurs in the rural population of geographically limited areas of Bulgaria, Bosnia and Herzegovina, Croatia, Romania, and Serbia, and a number of theories have been proposed about its aetiology. The mycotoxin theory has prevailed until now, based on the studies of nephrotoxic mycotoxin OTA that revealed higher frequency of OTA-positive food and blood samples in endemic than in non-endemic Balkan areas. However, recently it has been suggested that aristolochic acid is the aetiological cause of endemic nephropathy, because of the histological similarities in kidney lesions between patients suffering from endemic nephropathy and patients suffering from Chinese herbs nephropathy caused by aristolochic acid. Until now it has not been unequivocally proved that the inhabitants of endemic nephropathy areas are exposed to higher concentration of aristolochic acid than in other regions and the exposure pathways are rather uncertain. There is a paradox between the wide distribution of OTA throughout the world with the expected risk of OTA causing human renal disease and the rarity of reported cases demonstrating its role in chronic renal disease [32]. OTA and probably other mycotoxins could be major environmental factors in the occurrence of renal disease especially in developing countries according to Tatu and co-workers [40]. Some studies have found a geographic co-variation between OTA content in food/feed and Balkan endemic nephropathy manifestation; others have not [7]. The Romanian group [40] mention two actually competing theories attempting to explain the cause of Balkan endemic nephropathy: (1) the mycotoxin hypothesis, which considers that Balkan endemic nephropathy is produced by ochratoxin A ingested intermittently in small amounts by the individuals in the endemic regions, and (2) the Pliocene lignite hypothesis, which proposes that the disease is caused by long-term exposure to polycyclic aromatic hydrocarbons and other toxic organic compounds leaching into the well drinking water from low rank coals underlying or proximal to the endemic settlements. In this review we have outlined the current knowledge on ochratoxins. Future studies need to focus on possible factors and co-factors involved in aetiology of OTA renal disease and cancer.
